# Chaos game representation for comparison of whole genomes

**DOI:** 10.1186/1471-2105-7-243

**Published:** 2006-05-05

**Authors:** Jijoy Joseph, Roschen Sasikumar

**Affiliations:** 1 Computational Modelling and Simulation, Regional Research Laboratory (CSIR), Thiruvananthapuram, 695019, India

## Abstract

**Background:**

Chaos game representation of genome sequences has been used for visual representation of genome sequence patterns as well as alignment-free comparisons of sequences based on oligonucleotide frequencies. However the potential of this representation for making alignment-based comparisons of whole genome sequences has not been exploited.

**Results:**

We present here a fast algorithm for identifying all local alignments between two long DNA sequences using the sequence information contained in CGR points. The local alignments can be depicted graphically in a dot-matrix plot or in text form, and the significant similarities and differences between the two sequences can be identified. We demonstrate the method through comparison of whole genomes of several microbial species. Given two closely related genomes we generate information on mismatches, insertions, deletions and shuffles that differentiate the two genomes.

**Conclusion:**

Addition of the possibility of large scale sequence alignment to the repertoire of alignment-free sequence analysis applications of chaos game representation, positions CGR as a powerful sequence analysis tool.

## Background

Chaos game representation was proposed as a scale-independent representation for genomic sequences by H.J. Jeffrey [1]. A CGR of a DNA sequence is plotted in a unit square, the four vertices of which are labelled by the nucleotides A-(0,0), C-(0,1), G-(1,1), T-(1,0). The plotting procedure can be described by the following steps: the first nucleotide of the sequence is plotted halfway between the centre of the square and the vertex representing this nucleotide; successive nucleotides in the sequence are plotted halfway between the previous plotted point and the vertex representing the nucleotide being plotted.

Mathematically coordinates of the successive points in the chaos game representation of a DNA sequence is described by an iterated function system defined by the following equations

Xi=0.5 (Xi−1+gix)Yi=0.5 (Yi−1+giy)}     (1)
 MathType@MTEF@5@5@+=feaafiart1ev1aaatCvAUfKttLearuWrP9MDH5MBPbIqV92AaeXatLxBI9gBaebbnrfifHhDYfgasaacH8akY=wiFfYdH8Gipec8Eeeu0xXdbba9frFj0=OqFfea0dXdd9vqai=hGuQ8kuc9pgc9s8qqaq=dirpe0xb9q8qiLsFr0=vr0=vr0dc8meaabaqaciaacaGaaeqabaqabeGadaaakeaadaGacaqaauaabeqaceaaaeaacqqGybawdaWgaaWcbaGaeeyAaKgabeaakiabg2da9iabicdaWiabc6caUiabiwda1iaaykW7cqGGOaakcqqGybawdaWgaaWcbaGaeeyAaKMaeyOeI0IaeGymaedabeaakiabgUcaRiabbEgaNnaaBaaaleaacqqGPbqAcqqG4baEaeqaaOGaeiykaKcabaGaeeywaK1aaSbaaSqaaiabbMgaPbqabaGccqGH9aqpcqaIWaamcqGGUaGlcqaI1aqncaaMc8UaeiikaGIaeeywaK1aaSbaaSqaaiabbMgaPjabgkHiTiabigdaXaqabaGccqGHRaWkcqqGNbWzdaWgaaWcbaGaeeyAaKMaeeyEaKhabeaakiabcMcaPaaaaiaaw2haaiaaxMaacaWLjaWaaeWaaeaacqaIXaqmaiaawIcacaGLPaaaaaa@58FF@

where g_ix _and g_iy _are the X and Y co-ordinates respectively, of the corners corresponding to the nucleotide at position i in the sequence. For example if the i^th ^nucleotide is C,

g_ix _= 0 and g_iy _= 1.

CGRs of DNA sequences were shown to exhibit interesting patterns. These interesting features relevant to the DNA sequence organization attracted immediate further research [2-4]. CGR has been used in various kinds of investigations of DNA sequences. The first potential of CGR to be recognized was its capability to depict genomic signatures. Hill et al. [3] examined the CGRs of coding sequences of 29 relatively conserved alcohol dehydrogenase genes from phylogenetically divergent species. They found that CGRs were similar for the genes of the same or closely related species but were different for the genes from distantly related species. Oliver et al. [4] used the density of CGR points to derive entropy profiles for DNA sequences that showed a different degree of variability within and between genomes. Using CGR for making, oligomer frequency counts Deshavanne et al. [5] observed that subsequences of a genome exhibit the main characteristics of the whole genome, attesting to the validity of a genomic signature concept.

CGR research received a setback when Goldmann [6] asserted that simple Markov Chain models based solely on di-nucleotide and tri-nucleotide frequencies can completely account for the complex patterns exhibited in CGRs of DNA sequences. However Almeida et al. [7] showed that Markov chain models are in fact particular cases of CGRs. They showed that the distribution of points in CGR is a generalization of Markov chain probability tables that accommodates non-integer orders. Wang et al. [8] proved that while nucleotide, di-nucleotide and tri-nucleotide frequencies are able to influence the patterns in CGRs these frequencies cannot solely determine the patterns in CGRs. They showed that CGR is completely determined by frequencies of oligonucleotides of all lengths. The work of Almeida et al. positioned CGR as a powerful sequence modelling tool that has the advantages of computational efficiency and scale independence.

Sequence comparisons can be made using two different aspects of the CGR:

1. The frequency matrices of oligonucleotides of different lengths, that are derivable from CGR by resolving the CGR using grids of different sizes

2. The co-ordinates of the CGR points of the two sequences

In the CGR, a point corresponding to a sequence of length 'n' is contained within a square with side of length 2^-n ^[1]. The frequency of appearance of any oligomer in a sequence can be found out by partitioning the CGR space into squares of appropriate sizes. Thus counting the CGR points in the squares of a 2^n ^× 2^n ^grid gives the number of occurrences of all possible n-mers in the sequence. This representation is called Frequency chaos game representation (FCGR) where frequency of an oligomer is the number of points in the corresponding square. It is also possible to calculate oligonucleotide frequencies of non-integer lengths by resolving the CGR using grids of sizes other than powers of two. [7]

Most applications of CGR have been based on point counts calculated at various grid resolutions (FCGR). Sequence comparisons based on CGR co-ordinates have been left relatively unexplored. Almeida et al. [7], pointed out that, regions of local similarity between two sequences is reflected in the distance between CGR points. CGR points come closer together as sequence similarity increases. They defined a measure of local similarity as length of similar sequence n_H _calculated as a function of the maximum absolute difference between either CGR coordinate. However no attempt was made to use the information for developing an algorithm for aligning and comparing whole genomes.

As more and more genomes are being sequenced it has become possible to study evolutionary events by comparing whole genomes of closely related species and identifying the differences. Efficient programs for detecting and aligning matching segments in pairs of mega-base scale sequences is important for comparing whole genomes and determining evolutionary relationships. Several programs for large-scale genome comparison have been developed in the last six years, for example, MUMMER[9], SSAHA[10], AVID[11], BLASTZ[12]. All these programs follow an anchor-based approach in which all matching n-mers for a fixed n are first identified as potential anchors and the anchors are extended into longer alignments.

In this paper we develop a fast algorithm for comparison of pairs of long sequences using the information contained in CGR points. We first show how all similar segments of two sequences can be identified based on the distance between the CGR points of the two sequences. Since determination of distance between all pairs of CGR points, is costly in time (complexity O(N × M), N and M being the length of the two sequences), we speed up the program by using an anchored alignment approach similar to that used in other programs. We use CGR resolved by a 2^n ^× 2^n ^grid for fast location of the matching n-mers which form the anchors. The distance between CGR points corresponding to each pair of matching n-mers, is then used to see if the matching n-mers can be extended into longer local alignments. We allow for mismatches by chaining together close local alignments. The program finds multiple local alignments between two sequences, allowing the detection of homologous segments, internal sequence duplications and shuffling of segments.

## Results

Figures 3, 4, 5, 6, 7 show dot matrix plots showing the local alignments between Human Immunodeficiency Virus [GenBank: K02013.1] and Chimpanzee Immunodeficiency Virus [EMBL:X52154], Pyrococcus abyssi GE5 [EMBL:AL096836] and Pyrococcus horikoshii OT3 [DDBJ:BA000001], E. coli OH157:H7 [DDBJ:BA000007] and E. coli K12 [GenBank: U00096], Rickettsia p. madrid E. [EMBL:AJ235269] and Rickettsia c. malish 7 [GenBank:AE006914], Mycobacterium leprae TN [EMBL:AL450380] and Mycobacterium tuberculosis H37Rv [EMBL:AL123456] respectively. It can be seen that large segments have been inverted between Pyrococcus abyssi and Pyrococcus horikoshii as well as between Mycobacterium leprae TN and Mycobacterium tuberculosis H37Rv. Text files giving positions of Insertions/Deletions and mismatches inferred from the local alignments between the two strains of E. coli are given as Supplementary material.

The time taken for finding all local alignments between pairs of sequences of different sizes is given in Table 1. It can be seen that the time of execution of the program depends not only on the length of the sequences, but also on the degree of similarity between them. For example, the time taken for comparing M.leprae and M.tuberculosis is much greater than the time taken for comparing M. bovis and M.tuberculosis even though the sizes of the genomes are similar. The time taken by this program for comparing the two E.Coli genomes is 68 seconds while MUMmer a large-scale sequence alignment tool available takes only 17 seconds. The emphasis of this paper is on the theoretical development of the method rather than on software development and it is possible that with better programming inputs the implementation can be made more efficient and faster. The main advantage of this method comes from the fact that CGR simultaneously facilitates other types of sequence comparisons ranging from visual comparisons of patterns to oligonucleotide frequency spectrums and genome signatures.

## Conclusion

A new algorithm that uses information from chaos game representation of genome sequences for finding all local alignments between the sequences has been developed. Fast comparisons can be made between sequences of megabase size using a Pentium IV machine. As far as the speed of alignment is concerned, the program, in its present state does not offer any major improvements over MUMmer, but it is possible that the method can be implemented more efficiently through better programming inputs. Addition of the possibility of large scale sequence alignment to the existing repertoire of alignment-free sequence analysis possibilities from chaos game representation, positions CGR as a powerful quantitative sequence analysis tool.

## Methods

### Using CGR points for finding identical segments in two sequences

In the following we show how the distance between CGR points can be used to identify sequence identities without having to match the sequences nucleotide by nucleotide.

Consider the i^th ^nucleotide of one sequence and the j^th ^nucleotide of the other. Co-ordinates of the CGR points corresponding to these positions on the two sequences are given by:

X_i _= 0.5 (X_i-1_+g_ix_)     Y_i _= 0.5 (Y_i-1_+g_iy_)

X_j _= 0.5 (X _j-1_+g_jx_)     Y_j _= 0.5 (Y _j-1_+g_jy_)     (2)

The distance between CGR points is defined as

*d*(*i*, *j*) = max(*abs*(*X*_*i*_*- X*_*j*_), *abs*(*Y*_*i *_- *Y*_*j*_))     (3)

If the nucleotides at positions 'i' and 'j' of the first and the second sequence respectively are equal then g_ix _= g_jx _and g_iy _= g_jy _Then from equations (2) and (3) we get,

*d*(*i*, *j*) = 0.5 *d*(*i *- 1, *j *- 1)     (4)

i.e. A pair of similar nucleotides makes the distance between the corresponding CGR points, half the distance between the previous pair of points. Extending this argument, we can say that if k consecutive nucleotides previous to positions i and j on the two sequences are identical, the distance between the CGR points corresponding to i and j is given by

*d*(*i*, *j*) = (0.5)^*k *^*d*(*i *- *k*, *j *- *k*)     (5)

As k increases d (i, j) becomes smaller, i.e. as the length of identical sequence increases, the CGR points come closer together.

It must be noted that the closeness of two CGR points is not a sufficient condition to conclude that there is a length of similar sequence behind them. d (i, j) can become very low even when the sequences are very different. Such cases correspond to points on either side of, but close to, the borders of the quadrants corresponding to the four nucleotides. However if eqn. (5) is satisfied it can be inferred that the sequence segment (i-k to i) in one sequence is identical to the segment (j-k to j) in the other sequence.

Taking log on both sides of eqn. (5), we get,

k=log⁡(d(i,j))−log⁡(d(i−k,j−k))log⁡(0.5)     (6)
 MathType@MTEF@5@5@+=feaafiart1ev1aaatCvAUfKttLearuWrP9MDH5MBPbIqV92AaeXatLxBI9gBaebbnrfifHhDYfgasaacH8akY=wiFfYdH8Gipec8Eeeu0xXdbba9frFj0=OqFfea0dXdd9vqai=hGuQ8kuc9pgc9s8qqaq=dirpe0xb9q8qiLsFr0=vr0=vr0dc8meaabaqaciaacaGaaeqabaqabeGadaaakeaacqWGRbWAcqGH9aqpdaWcaaqaaiGbcYgaSjabc+gaVjabcEgaNjabcIcaOiabdsgaKjabcIcaOiabdMgaPjabcYcaSiabdQgaQjabcMcaPiabcMcaPiabgkHiTiGbcYgaSjabc+gaVjabcEgaNjabcIcaOiabdsgaKjabcIcaOiabdMgaPjabgkHiTiabdUgaRjabcYcaSiabdQgaQjabgkHiTiabdUgaRjabcMcaPiabcMcaPaqaaiGbcYgaSjabc+gaVjabcEgaNjabcIcaOiabicdaWiabc6caUiabiwda1iabcMcaPaaacaWLjaGaaCzcamaabmaabaGaeGOnaydacaGLOaGaayzkaaaaaa@59DD@

We can get an upper bound for k by putting d(i-k, j-k) = 1 in eqn.(6):

*k*_max _= -log_2 _(*d*(*i*, *j*))     (7)

This can be seen to be the same as the length of similar sequence proposed by Almeida et al. as a measure for assessing local similarity in two sequences.

Equations 5 and 7 can be used to develop an algorithm for detection of all identical segments in two sequences based on the distance between CGR points.

**Figure 1:**
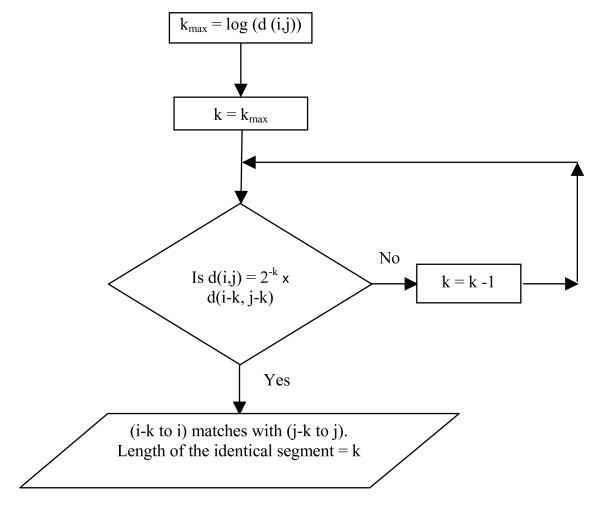
**Finding k**. Flow chart of the procedure for identifying matching segments

Calculating k_max _for a pair of positions (i, j) on the two sequences we can estimate that, at the most, the sequence segment from i to i- k_max _in one sequence could be identical to the segment from j to j- k_max _in the other sequence. We then check whether eqn. (5) is satisfied for k = k_max _to see if these segments are truly identical. If not, we substitute k-1 for k and again check again if eqn.(5) is satisfied and if not, the procedure is repeated till the condition is satisfied. Thus starting from (i- k_max_, j- k_max_), the first position (i-k, j-k) that satisfies eqn.(5) is determined. This gives the length k up to which segments prefixed to positions i and j in the two sequences, are identical. The flow chart of this procedure is shown in Fig. 1

This method thus identifies identical segments without having to match the whole segment nucleotide by nucleotide. Search can be completely avoided if k_max _is found to be less than a threshold and long homologous segments can be identified by checking only a few points from (i- k_max_, j- k_max_) instead of matching the whole length of the segment.

### Speeding up the algorithm

The disadvantage of the above method is that the computational cost is of the order of the product of the length of the two sequences.

In order to speed up the program, we find a way to avoid computing d(i, j) for all pairs of CGR points of the two sequences. For this we use information from a resolved CGR in which the CGR square is divided into grid of size 2^n ^× 2^n^. All CGR points falling in a square denotes the existence of a particular n-mer prefixed to that position.

The algorithm for comparing two sequences A and B is described below:

1. The CGR co-ordinates for both the sequences are calculated

2. The CGR is resolved using a 2^n ^× 2^n ^grid and the CGR points of sequence B that fall in each square are noted and stored

3. Starting from the last nucleotide of sequence A, we identify the square in which the corresponding CGR point i falls.

3. The CGR points of B that fall in the same square, correspond to the n-mers in B that match the n-mer which is prefixed to the position i in A

4. We calculate d (i, j) and k_max _for those CGR points j of the sequence B, which fall in the same square as the CGR point i of sequence A.

5. Using d(i, j) and k_max_, we determine the length of matching segments, as described in the last section (Fig.1)

6. The longest matching segment is taken as the best local alignment at position i

7. The procedure is repeated next for the point i-k in A, k being the length of the longest matching segment.

We can thus find all the non-overlapping local alignments between the two sequences. Using this approach, the all- to-all comparisons of the previous section is reduced to some-to-some comparisons, which speeds up the algorithm considerably. This technique is similar to the anchored alignment method used in other alignment programs; the difference is that we use information from CGR, both for finding the anchors as well as for extending them.

The program yields the list of all local alignments between the two sequences in the order of their position in the sequence A. An example list of alignments is shown in Table 2.

### Floating point error

For long identical sequence segments, the distance value may go below the minimum value possible for a floating point variable. The distance defined in double precision variable becomes zero when the length of identical segment is greater than 64.

Therefore in our implementation, when we encounter zero value for the distance we jump back by sixty positions and check distance again; if the distance is again zero, we jump back another sixty positions and so on until the distance becomes non-zero. We add all the skipped positions to the k that we finally calculate with the non-zero distance value.

### Analysing the local alignments for shuffles, mismatches and insertion/deletions

**Figure 2:**
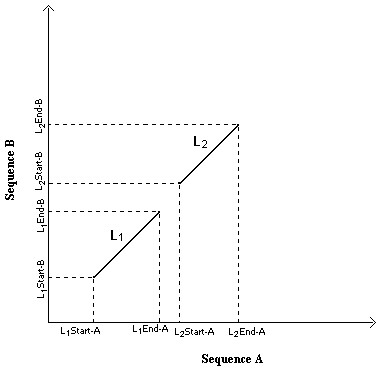
**Local alignments**. Definition of local alignments

**Figure 3:**
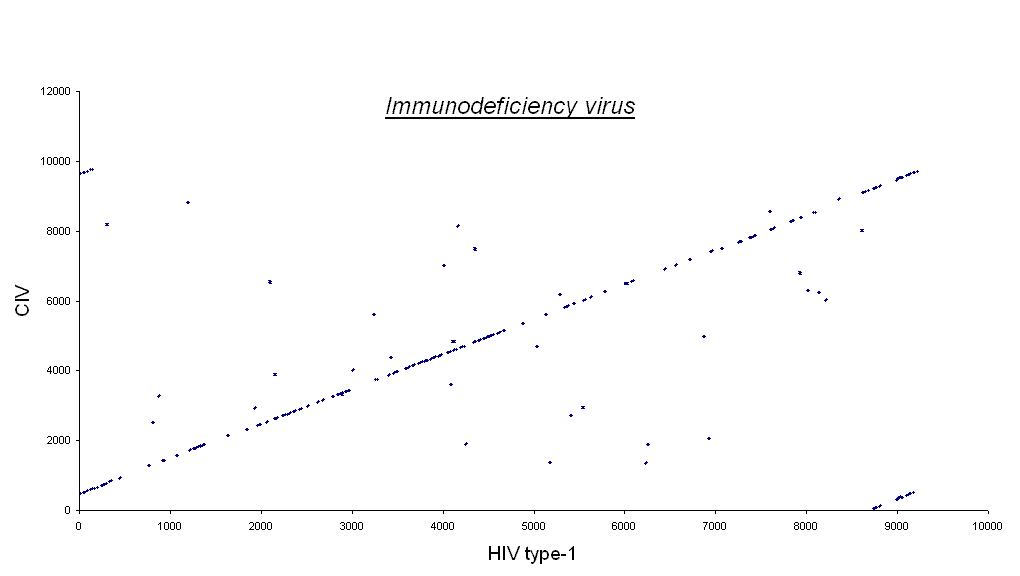
**Immunodeficiency virus**. Human immunodeficiency virus and Chimpanzee immunodeficiency virus

**Figure 4:**
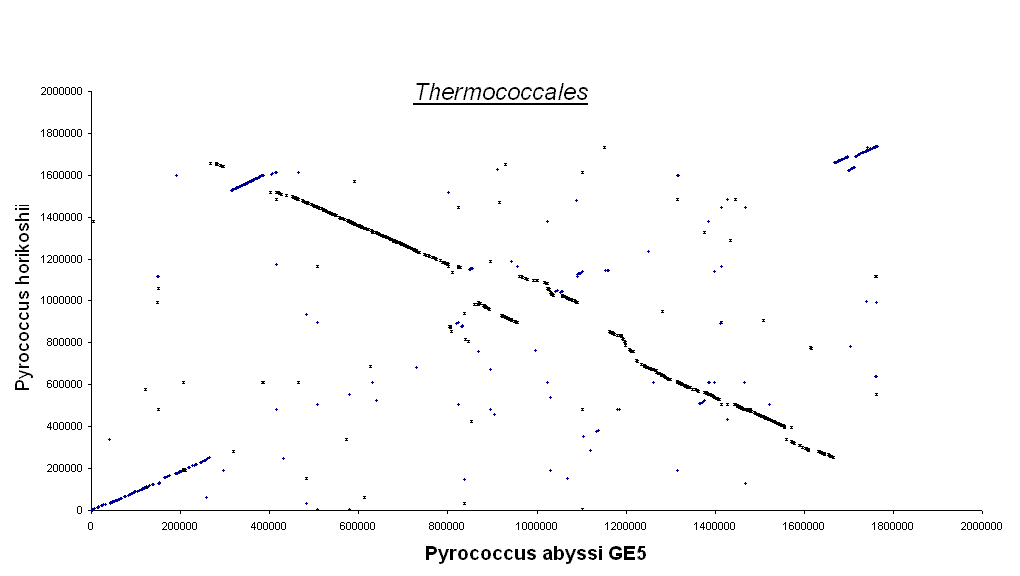
**Thermococcales**. Pyrococcus Abyssi and Pyrococcus Horikoshii

**Figure 5:**
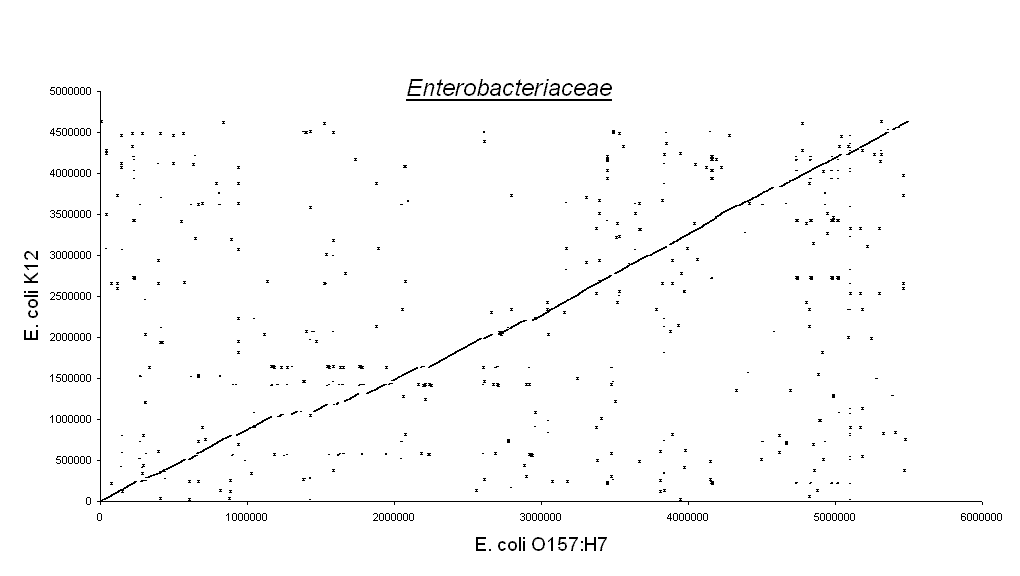
**Enterobacteriaceae**. E.coli K12 and E.coli O157:H7

**Figure 6:**
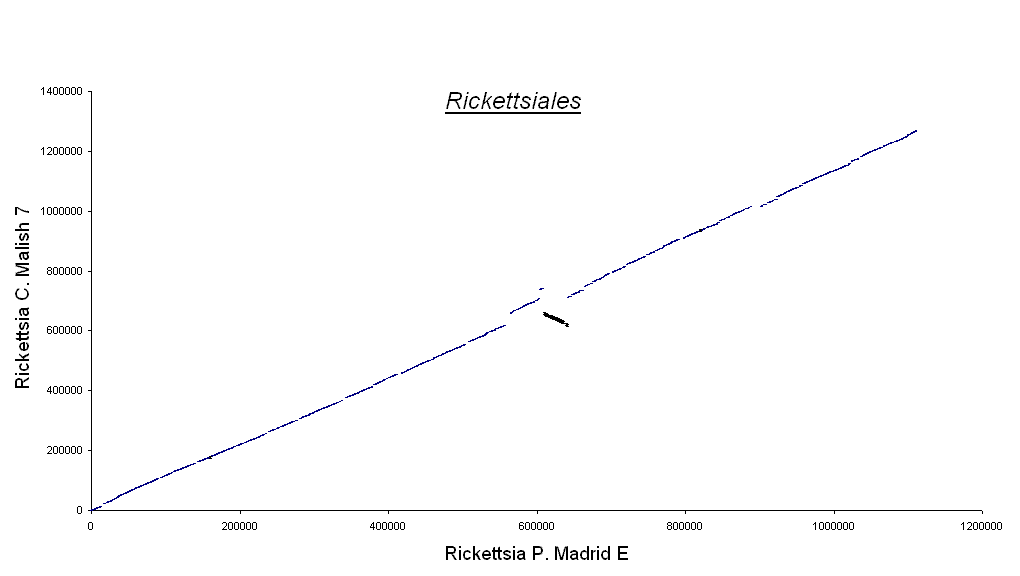
**Rickettsiales**. Rickettsia Prowazekii Madrid E and Rickettsia Conorii Malish 7

**Figure 7:**
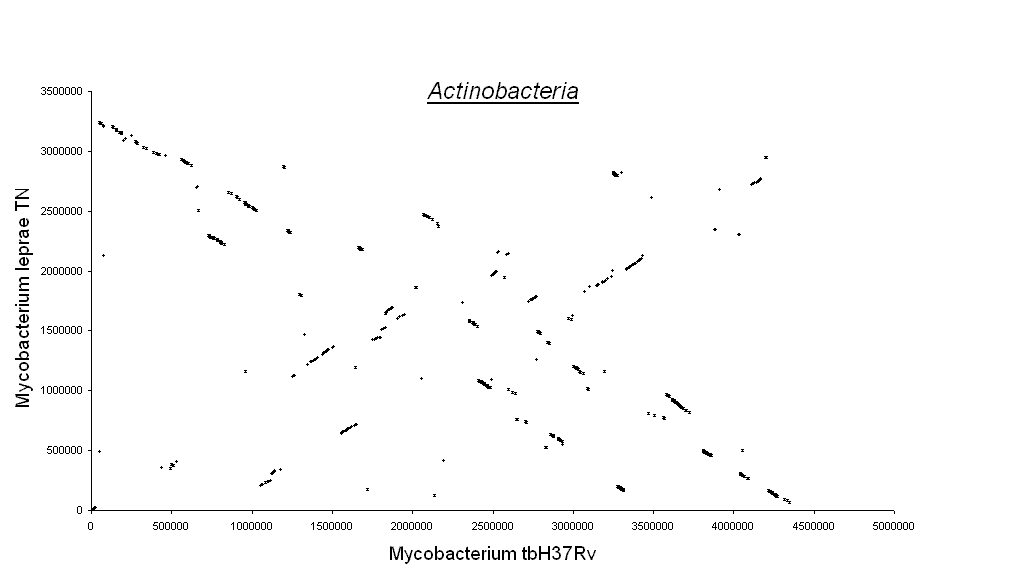
**Actinobacteria**. Mycobacterim Tuberculosis H37Rv and Mycobacterium Leprae TN

A local alignment can be defined by the start and end positions of identical segments in the two sequences. A pair of local alignments is given in figure 2.

Consider two local alignments L_1 _and L_2 _defined by

(L_1_.START.A, L_1_.END.A, L_1_.START.B, L_1_.END.B) and

(L_2_.START.A, L_2_.END.A, L_2_.START.B, L_2_.END.B)

#### (a) Shuffles/Rearrangements

Consider the list of local alignments that are ordered in increasing order of L.END.A. This list may not be in increasing order of L.END.B and any disruption of order in the list with respect to position in Sequence B is indicative of shuffling. By examining the disruption of order in L.END.B we can estimate the number of shuffles that have taken place in Sequence B with respect to Sequence A.

#### (b) Mismatches

Let, ΔA = abs (L_2_.START.A – L_1_.END.A) and

ΔB = abs (L_2_.START.B – L_1_.END.B)

where L_1 _and L_2 _are two consecutive alignments in the ordered list.

Mismatch length between the alignments can be calculated as:

Mismatch length = min (ΔA, ΔB)

Mismatches between the forward strands of the genomes of E.coli K12 and E.coli O157:H7 is given as additional file [see Additional file 1].

#### (c) Insertions/Deletions

Diagonal off-set between two consecutive local alignments that are consecutive in Sequence B also, indicate deletions and insertions and can be calculated as

IN/DEL length = max (ΔA, ΔB)-min(ΔA, ΔB)

Insertions/Deletions between the forward strands of the genomes of E.coli K12 and E.coli O157:H7 is given as additional file [see Additional file 2].

#### (d) Duplications

Duplications in B can be identified wherever L_1_.START.A=L_2_.START.A and L_1_.END.A = L_2_.END.A

#### (e) Inversions

Inversion of segments is detected by finding local alignments between Sequence A and the reverse complement of Sequence B

### Chaining local alignments and filtering background noise

Short spurious alignments or background noise can be removed by filtering out all alignments below a certain threshold length. However this carries with it the danger of filtering out many "true" alignments that are separated by small mismatches. Therefore before filtering it is better to chain together the perfect local alignments by allowing a certain amount of mismatches. We allow for short mismatches by chaining together local alignments that are have no diagonal off-set and differ only by mismatches of a few nucleotides. We specify the maximum allowable mismatches per length of the chained alignment. If there is no diagonal off-set between them i.e. ΔA = ΔB, and the mismatch falls below the threshold value, the two alignments are chained together into a single alignment.

Chained alignments having length below a threshold are discarded to filter out the background noise. Text file showing matching regions between the genomes of E.coli K12 and E.coli O157:H7, after filtering background noise, is given as additional file [see Additional file 3]. Further, the forward strand of E.coli K12 is compared with the complementary strand of E.coli O157:H7. The resulting text file showing the matching regions is given as additional file [see Additional file 4].

## Availability and requirements

The source code for finding matching segments using CGR is given as additional file [see Additional file 5].

The source code for chaining matching segments and filtering background noise and also showing insertions/deletions/mismatches is given as additional file [see Additional file 6]

Operating system: Linux

Programming language: Standard C

License: GNU General Public License

## Abbreviations

CGR – Chaos Game Representation

FCGR – Frequency Chaos Game Representation

## Authors' contributions

RS developed the relationship between the length of identical segments and distance between CGR points. JJ developed the algorithm for speeding up the comparison. JJ did all the coding and generated the results. RS wrote the paper and JJ made editorial corrections.

## Supplementary Material

Additional File 1 Text file showing mismatches between the forward strands of the genomes of E.coli K12 and E.coli O157:H7. Click here for file

Additional File 2 Text file showing insertions/deletions between the forward strands of the genomes of E.coli K12 and E.coli O157:H7. Click here for file

Additional File 3 Text file showing matching segments in forward strands of both genomes of E.coli K12 and E.coli O157:H7. Click here for file

Additional File 4 Text file showing matching segments in the forward strand of E.coli O157:H7 reverse strand of E.coli K12 (inversions). Click here for file

Additional File 5 Source code of the program for finding similar sequences in two sequences. Click here for file

Additional File 6 The Source code of the programme for chaining aligned segments, filtering background noise and for identifying insertions deletions and mismatches. Click here for file

## Figures and Tables

**Table 1:** Computational time chart. Table 1 shows the computation time taken by the program running on a Pentium IV 2.5 GHz machine, for comparing various genome sequences.

**Organisms**	**Length A (In base pairs)**	**Length B (In base pairs)**	**Time **(forward strand) **in seconds**	**Time **(reverse complement) **in seconds**
HIV vs CIV	9229	9811	<1	1
P. Abyssi vs P. Horikoshii	1765118	1738505	24	27
E. coli O157:H7 vs E. coli K12	5498450	4639675	68	156
R. p. Madrid E vs R. c. Malish 7	1111523	1268755	18	24
M. tuberculosis H37Rv vs M. leprae TN	4411532	3268203	119	120
M. bovis AF2122 vs M.tuberculosis H37Rv	4345492	4411532	10	230
M. tuberculosis H37Rv vs M. tuberculosis CDC1551	4411532	4403662	6	232

**Table 2:** Sample alignment list.A sample list of all local alignments between two sequences in the order of their position in the sequence A is shown in Table 2:

**Order in A**	**StartA**	**EndA**	**Order in B**	**StartB**	**EndB**	**Length**
0	8127	8158	0	9402	9433	31
1	10846	10920	1	12193	12267	74
2	11125	11158	2	12446	12479	33
3	18260	18296	3	20577	20613	36
4	20041	20109	4	22402	22470	68
5	20923	20975	5	23284	23336	52
6	21233	21284	6	23594	23645	51
7	23591	23622	7	25970	26001	31
8	26750	26835	8	28887	28972	85
9	53377	53437	9	37193	37253	60
10	67041	67072	84	493374	493405	31
11	67200	67231	85	493533	493564	31
12	143809	143840	262	2932826	2932857	31
---	------	------	-------	-------	-------	-------
